# Oral Ketorolac with Inferior Alveolar Nerve Block for Irreversible Pulpitis: A Systematic Review and Meta-analysis

**DOI:** 10.2174/1874210601812010340

**Published:** 2018-04-30

**Authors:** Gowri Sivaramakrishnan, Kannan Sridharan

**Affiliations:** 1 Department of Oral Health, College of Medicine, Nursing and Health Sciences, Fiji National University, Brown Street, Suva, Fiji; 2 Department of Pharmacology and Therapeutics, College of Medicine and Medical Sciences, Arabian Gulf University, Manama, Bahrain

**Keywords:** Ketorolac, Pre-medication before IANB, Irreversible pulpitis, Pain, Root canal treatment, Endodontics

## Abstract

**Background:**

Ketorolac has advantages over other analgesics as a pre-anaesthetic medication. Considering this in mind, the present meta-analysis aims to identify the effect of oral ketorolac premedication on the anaesthetic efficacy of Inferior Alveolar Nerve Block (IANB) in patients with irreversible pulpitis.

**Methods:**

Full-texts of eligible studies were obtained from electronic databases. The extracted data was analysed using non-Cochrane mode in RevMan 5.0 software. Relative risk [95% CI] was calculated for the success of IANB.

**Results:**

Four studies were included for the final review. The success rate of IANB on 221 patients with relative risk of 1.87 [1.36, 2.56] was statistically significant favouring ketorolac. The mean difference for VAS in 171 patients was not statistically significant {-13.55 [-33.91, 6.82]}.

**Conclusion:**

Oral ketorolac can be successfully administered as a premedication before conventional inferior alveolar nerve block for endodontic treatment for irreversible pulpitis.

## 1 INTRODUCTION

Management of pain is one most important aspect to be addressed while planning treatment procedures in dentistry, especially endodontic treatment. Inferior Alveolar Nerve Block (IANB) has been routinely used to achieve profound anaesthesia during endodontic treatment. However, the success rate of IANB alone has been reported to vary between 15 and 57% [1]. The practice of prescribing pre-medication using various Non-Steroidal Anti-Inflammatory Drugs (NSAIDs) and opioid analgesics for pulpal inflammation, prior to local anaesthesia, has been explored in various randomised controlled trials [2-5]. NSAIDs act by inhibiting cyclooxygenase and recently scavenging of reactive intra-cellular products has been attributed for its anti-inflammatory activity [6]. Amongst the NSAIDs, ketorolac has beneficial effects in patients with exaggerated or severe inflammation and it can be used even as an opioid-sparing analgesic drug [7]. Parenteral ketorolac has been suggested as an appropriate analgesic in the pre-hospital settings in patients with acute pain [8]. Patients with irreversible pulpitis have moderate to severe type of odontogenic pain consequent to which the success of IANB may be low. In a recent study, it was observed that pulpitis patients without pain have better success rate for IANB than with pain [9]. Hence, various investigators have tried ketorolac as a pre-anaesthetic medication for improving success of IANB. However, until date, there is no systematic compilation on such studies to observe the relative effect estimate of ketorolac with other pre-anaesthetic drugs. Thus, we carried out the present systematic review and meta-analysis to identify the anaesthetic efficacy of inferior alveolar nerve block after pre-medication with oral ketorolac, for endodontic treatment of tooth diagnosed with irreversible pulpitis.

## 2 MATERIALS AND METHODS

### 2.1 Information Sources and Search Strategy

The protocol for this review was registered with International Prospective Register of Systematic Reviews (PROSPERO) with the registration number CRD42016039273. The review protocol can be accessed at http://www.crd.york.ac.uk/PROSPERO/register_new_review.asp?RecordID=39273&UserID=16309

A through literature search was conducted and completed on 20 May 2016 on Medline (*via *PubMed), Cochrane central register of clinical trials (CENTRAL) and Database of Abstracts of Reviews of Effects (DARE) using ketorolac [tiab] and inferior alveolar nerve block [tiab] as keywords. This search was further supplemented by hand searching of references from eligible studies. Studies published only in English language were included.

### 2.2 Eligibility Criteria

Only those studies with randomized controlled design with the following requirements were included in the present study:

1. Type of participants: Patients with irreversible pulpitis in any tooth and requiring endodontic treatment under inferior alveolar nerve block with lignocaine using conventional technique.
2. Type of intervention: Oral ketorolac pre-medication before conventional inferior nerve block with lignocaine.
3. Comparison: Placebo or any other oral pre-medication with any drug before inferior nerve block with lignocaine.
4. Outcome: The success rate of the Inferior Alveolar Nerve Block (IANB) and the assessment of pain measured with Visual Analog Scale (VAS).

### 2.3 Study Procedure

Two authors independently screened all the data and identified abstracts for possible inclusion. Full-text articles were obtained for all the eligible studies. A pre-tested data extraction form was created and both the authors independently extracted trial site, year, trial methods, participants, interventions, and outcomes. Disagreement between the authors was resolved through discussion. The extracted data were analysed using non-Cochrane mode in RevMan 5.0 software. The methodological quality of eligible trials was assessed using the Cochrane collaboration’s tool for assessing the risk of bias. We followed the guidance to assess whether trials took adequate steps to reduce the risk of bias across six domains: sequence generation, allocation concealment, blinding (of participants, personnel, and outcome assessors), incomplete outcome data, selective outcome reporting, and other sources of bias. The judgment was categorized into the low, high or unclear risk of bias [10]. Due to a limited number of studies, publication bias could not be checked. Percent difference between the experimental (ketorolac group) and control (placebo or any other drug) group were assessed and the mean difference in the percent and percent standard error were considered for final assessment. Relative risk [95% CI] was calculated for the success of IANB. The heterogeneity between the studies was assessed using the Forest plot visually, I^2^ statistics wherein more than 50% was considered to have moderate to severe heterogeneity and Chi-square test with a statistical *P*-value of less than 0.10 to indicate statistical significance. Random-effect models were used in case of moderate heterogeneity. The present meta-analysis was conducted and presented in accordance with Preferred Reporting Items for Systematic Reviews and Meta-Analyses (PRISMA) guidelines [11].

## 3 RESULTS

### 3.1 Search Results

**Fig. (1):**
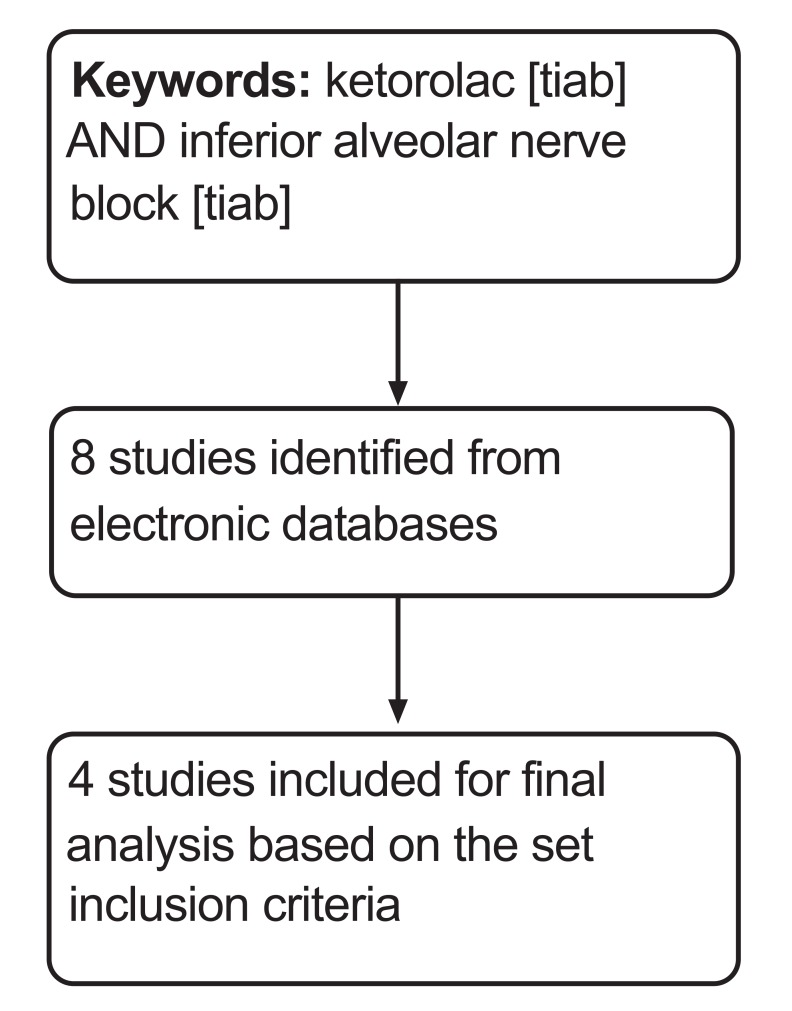
Study flow diagram.

Eight studies were obtained during the screening, of which four were found to be eligible to be included for the final review and quantitative synthesis (Fig. **1**). Table **1** lists the key characteristics of the included studies [12-15]. A summary of risk of bias of included studies is depicted in Fig. (**2**).

### 3.2 Pooled Results

#### 3.2.1 Success Rate of IANB

**Fig. (3):**
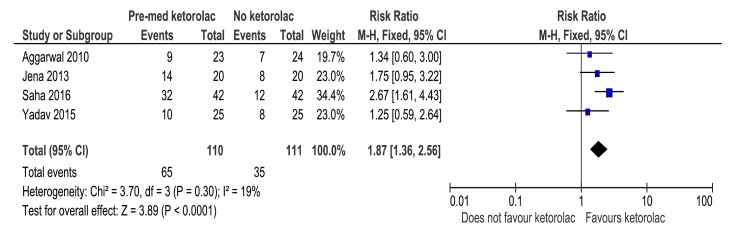
Forest plot of success rate of IANB.

All the four included studies evaluated the success rate of IANB with ketorolac pre-medication in comparison with placebo, in a total of 221 study participants in which 65 successful events were observed in the ketorolac group and 35 in the placebo group. The pooled relative risk for the success of block was observed to be 1.87 [1.36, 2.56] and was statistically significant favouring the use of ketorolac as a pre-anaesthetic medication (Fig. **3**).

#### 3.2.2 VAS

**Fig. (4):**
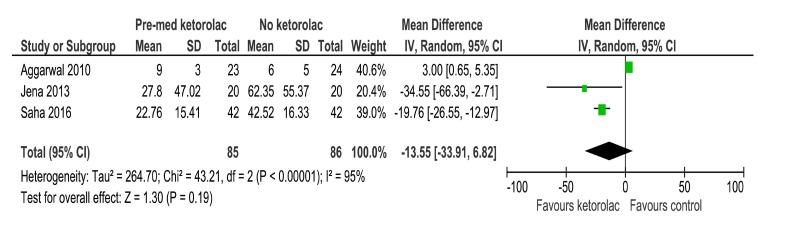
Forest plot of VAS.

Three out of four studies compared VAS for pain between the ketorolac and placebo group in a total of 171 patients. The pooled mean difference was not statistically significant between the groups with the MD being -13.55 [-33.91, 6.82] (Fig. **4**).

## 4 DISCUSSION

This systematic review aims to identify the anaesthetic efficacy of conventional inferior alveolar nerve block using lignocaine when pre-medication using oral ketorolac is administered.

Inferior alveolar nerve block using lignocaine and adrenaline has been routinely used to gain profound analgesia before endodontic treatment of teeth diagnosed with irreversible pulpitis. However, 90% of the practitioners experienced difficulties in achieving profound analgesia. In specific, a greater number of failures have been reported to occur with inferior alveolar nerve block [16] especially in teeth manifesting with irreversible pulpitis [17]. Various reasons reported for this failure include anxiety and fear, accessory innervation and concentration and volume of local anaesthetic and vasoconstrictor [18]. Keeping the failure rates in mind, studies focussed on using pre-medication with NSAID’s and steroids and identified the anaesthetic efficacy of inferior alveolar nerve block after pre-medication. A systematic review on the effect of premedication on inferior alveolar nerve block was compiled by Lapidius *et al*. However not all studies were included in the systematic analysis [19].

Pre-operative medication has been suggested as a measure to increase the efficacy of inferior alveolar nerve block. Various NSAID’s such as ibuprofen, ketorolac, diclofenac and steroids like dexamethasone have been tried in various randomised controlled trials [4, 5, 12]. Ketorolac has been a preferred due to its advantages over other NSAID’s. These include less likely alterations to the bleeding time, less likely to cause acute renal failure in patients with pre-existing renal impairment, less likely combination reactions when co-administered with other drugs and single dose administration [20]. Considering the advantages of ketorolac over other NSAID’s this systematic review and meta-analysis identified the anaesthetic efficacy of inferior alveolar nerve block after pre-medication with ketorolac. Apart from ketorolac, other non-steroidal anti-inflammatory drugs such as ibuprofen and acetaminophen (in combination) [21]. Recently, Praveen *et al.* [22] conducted a trial comparing ketorolac with prednisolone and the latter drug was observed with more persistent effect than ketorolac and placebo. Apart from oral administration, ketorolac has also been shown to be effective as buccal administration [23]. In contrast, adjuvant ketorolac with nitrous oxide has not been shown to have any additive analgesic effect in patients with symptomatic pulpitis [24].

The four studies included in the systematic review and meta-analysis identified ketorolac oral pre-medication had a statistically significant effect on the success rate of an inferior alveolar nerve block in patients with irreversible pulpitis. However, the pain scale as measured by VAS was not statistically significant. The study is limited in the fact that EMBASE could not be searched for appropriate studies. Nevertheless, checking the cross-references of the included studies is likely to negate this issue.

## CONCLUSION

Considering the advantages of ketorolac over other agents and the results obtained in the present study, we conclude from the systematic review and meta-analysis that oral premedication using ketorolac should be considered before conventional inferior alveolar nerve block for endodontic treatment of irreversible pulpitis. However, long-term randomized controlled trials are warranted.

## CONSENT FOR PUBLICATION

Not applicable.

## Figures and Tables

**Fig. (2):**
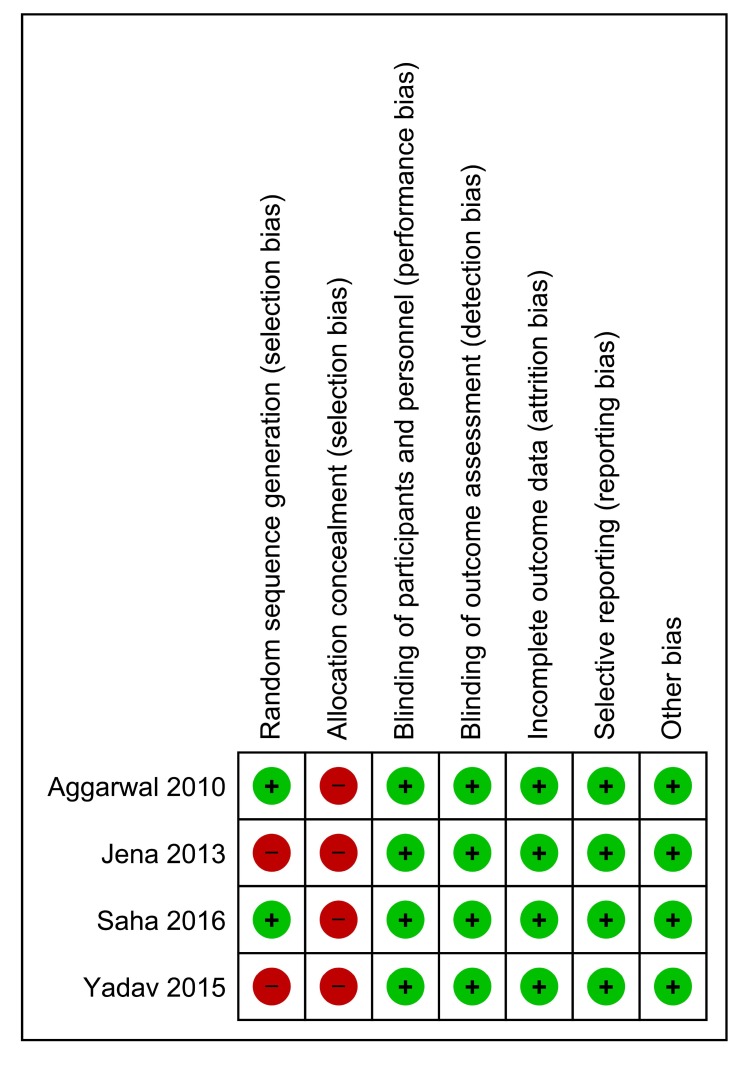
Summary of risk of bias.

**Table 1:** Key characteristics of the included studies.

**Author**	**Participants**	**Intervention**	**Control**	**Outcome**
Jena A 2013^8^	100 Adult patients requiring endodontic treatment for tooth diagnosed with irreversible pulpitis	20 patients-Oral ketorolac 10mg pre-medication after which conventional inferior alveolar nerve block will be administered.	20 patients-placebo with sugar coated pills 20 patients-ibuprofen 600 mg 20 patients-etodolac with paracetamol(400mg+500mg) 20 patients-aceclofenac with paracetamol(100mg+500mg) After all the above mentioned control, conventional inferior alveolar nerve block will be administered.	Visual analog scale Overall success of inferior alveolar nerve block
Ganguly SA 2016^9^	126 adult patients requiring endodontic treatment for tooth diagnosed with irreversible pulpitis	42 patients- ketorolac pre-medication after which conventional inferior alveolar nerve block will be administered	42 patients- diclofenac potassium 42 patients- Placebo After all the above mentioned control, conventional inferior alveolar nerve block will be administered.	Visual analog scale Overall success of inferior alveolar nerve block
Aggarwal V 2010^10^	62 adult patients requiring endodontic treatment for tooth diagnosed with irreversible pulpitis	24 patients- ketorolac 10mg pre-medication after which conventional inferior alveolar nerve block will be administered	24 patients- ibuprofen 300mg 24 patients- placebo After all the above mentioned control, conventional inferior alveolar nerve block will be administered.	Visual analog scale Overall success of inferior alveolar nerve block
Yadav M 2015^11^	50 adult patients requiring endodontic treatment for tooth diagnosed with irreversible pulpitis	25 patients -ketorolac 10mg pre-medication after which conventional inferior alveolar nerve block will be administered after buccal and lingual infiltrations.	25 patients- no pre-medication. Conventional inferior alveolar nerve block will be administered after buccal and lingual infiltrations.	Overall success of inferior alveolar nerve block
